# Cytosolic heat shock protein 90 is required for photoreceptor outer segment development and vision

**DOI:** 10.1016/j.jbc.2026.113192

**Published:** 2026-05-24

**Authors:** Hunter L. Aliff, Alexis B. Crockett, Daniella Munezero, Chyanne Reid, Hayley G. Bockius, Sierra G. Kuzak, Scott Rhodes, Thamaraiselvi Saravanan, Visvanathan Ramamurthy

**Affiliations:** 1 Departments of Biochemistry and Molecular Medicine, West Virginia University, Morgantown, West Virginia, USA; 2 Departments of Neuroscience, West Virginia University, Morgantown, West Virginia, USA; 3 Departments of Pharmaceutical and Pharmacological Sciences, West Virginia University, Morgantown, West Virginia, USA; 4 Departments of Ophthalmology and Visual Sciences, West Virginia University, Morgantown, West Virginia, USA

**Keywords:** Photoreceptors, heat shock proteins, hsp90, peripherin, PDE6, outer segment, vision, retinal degeneration, cytosolic hsp90

## Abstract

Protein chaperones, such as heat shock protein 90 (HSP90), play essential roles in proteostasis by stabilizing client proteins and facilitating proper folding. Evidence for HSP90 function in vision is substantiated by reports of visual disturbances in patients treated with pan-HSP90 inhibitors. Among the cytosolic HSP90 paralogs, loss of HSP90α permits normal photoreceptor development, outer segment (OS) morphogenesis, and function, but rods subsequently undergo progressive degeneration despite upregulation of the closely related paralog HSP90β. In contrast, cone function is preserved in the absence of HSP90α, raising the question of whether cytosolic HSP90 paralogs have redundant functions or display functional specialization between rods and cones. To address this question, we generated a conditional KO of HSP90β (*Hsp90ab1*), which revealed that loss of HSP90β alone does not disrupt photoreceptor development, morphology, or visual function. In contrast, cone-specific ablation of both cytosolic paralogs resulted in rapid loss of cone function, demonstrating a shared requirement for cytosolic HSP90 activity in cones. To examine potential redundancy during development, we generated mice with targeted deletion of both cytosolic HSP90 paralogs within the photoreceptor lineage. Although retinal lamination was preserved, photoreceptors failed to elaborate OS, and visual responses were abolished. The severe visual phenotype correlated with reduced levels of peripherin, a protein essential for OS morphogenesis. Together, the findings define stage- and cell type–specific requirements for cytosolic HSP90 paralogs in the retina and establish a central role for cytosolic HSP90 in photoreceptor OS development and function.

Protein homeostasis is essential for cell survival, and its disruption is a common feature of many neurodegenerative diseases, including retinal degenerative disorders that lead to blindness (1, 2). Protein chaperones such as heat shock protein 90 (HSP90) play central roles in proteostasis by stabilizing client proteins and facilitating proper folding (3, 4, 5, 6, 7, 8, 9). In clinical trials, treatment with pan-HSP90 inhibitors was associated with visual disturbances and night blindness (10, 11, 12). Consistent with these observations, studies in animal models showed that HSP90 inhibition compromises photoreceptor function, linking HSP90 activity to retinal biology (13, 14, 15).

Two highly homologous cytosolic HSP90 paralogs, HSP90α (*Hsp90aa1*) and HSP90β (*Hsp90ab1*), are expressed in most mammalian cells (4, 5, 16, 17). Despite their similarity, available evidence supports both overlapping and distinct functions for these paralogs (7, 9, 18). In previous work, we examined retinal structure and function in global HSP90α KO mice (HSP90α^−/−^). In these animals, photoreceptor development and early retinal organization were preserved. The visual function, along with photoreceptor outer segment (OS) morphogenesis, remained normal through postnatal day 45 (P45) (9). Beyond P45, rods exhibited a progressive decline in function accompanied by photoreceptor cell loss, indicating a requirement for HSP90α in long-term maintenance of mature rod photoreceptors, despite normal early development (9).

Further analysis of HSP90α-deficient retinas showed increased levels of HSP90β but reduced levels of rod phosphodiesterase 6 (PDE6), a key effector enzyme in the rod phototransduction cascade (9). These findings indicate that increased expression of HSP90β in the retina does not preserve rod PDE6 levels or photoreceptor function in the absence of HSP90α (9).

The high degree of homology between HSP90α and HSP90β suggests the possibility of functional redundancy during early photoreceptor development (7, 9, 16). In contrast, cone function is preserved in HSP90α KO mice, and HSP90β is preferentially expressed in cones (9, 14). These observations raise the possibility that cytosolic HSP90 paralogs may have overlapping roles during development but distinct or compensatory functions in mature photoreceptor subtypes, particularly in cones.

To address this question, we generated a mouse carrying conditional alleles of HSP90β (*Hsp90ab1*) using *Easi*-CRISPR/Cas9 technology (19, 20) and crossed them with animals expressing Cre recombinase in the outer retina, including photoreceptors. Loss of HSP90β alone did not disrupt photoreceptor development or function. To assess potential compensation by HSP90α in cones, we generated cone-specific double KOs of both paralogs using the human red/green pigment (HRGP) – Cre line and crossing it with HSP90α^−/−^ mice (21). In this context, cone function was abolished entirely, demonstrating a shared requirement for cytosolic HSP90 activity in cone photoreceptors.

To further examine compensation between HSP90 paralogs during photoreceptor development, we generated mice lacking both cytosolic HSP90 paralogs in the retina. The Cre recombinase expression was driven by a bacterial artificial chromosome (BAC) promoter for the *Crx* homeobox gene (22). In this BAC-*Crx*-Cre: HSP90^−/−^ model, retinal lamination was preserved, but photoreceptors failed to elaborate OSs. OS morphogenesis depends on proteins such as peripherin, which was markedly reduced in this model, leading to rapid degeneration and loss of photoreceptor function.

Taken together, our findings define stage- and cell type–specific requirements for cytosolic HSP90 paralogs in the retina. During early photoreceptor development, particularly OS morphogenesis, either paralog is sufficient. In mature rods, HSP90α is required for long-term maintenance. In cones, loss of either paralog alone is tolerated, whereas simultaneous removal of both paralogs results in rapid loss of cone function, indicating a shared requirement for cytosolic HSP90 activity in cones.

## Results

### Design and confirmation of HSP90β conditional KO mouse

Our previous work established an essential role for HSP90α in rod photoreceptor maintenance, while cone photoreceptors remained unaffected in an HSP90α KO model (9). Because protein expression patterns indicated enrichment of HSP90β in cones, we hypothesized that HSP90β may contribute to cone photoreceptor function. Because global germline deletion of *Hsp90ab1* is embryonically lethal, we developed a conditional KO model to investigate the cell-specific role of HSP90β within the retina (18).

**Figure 1:**
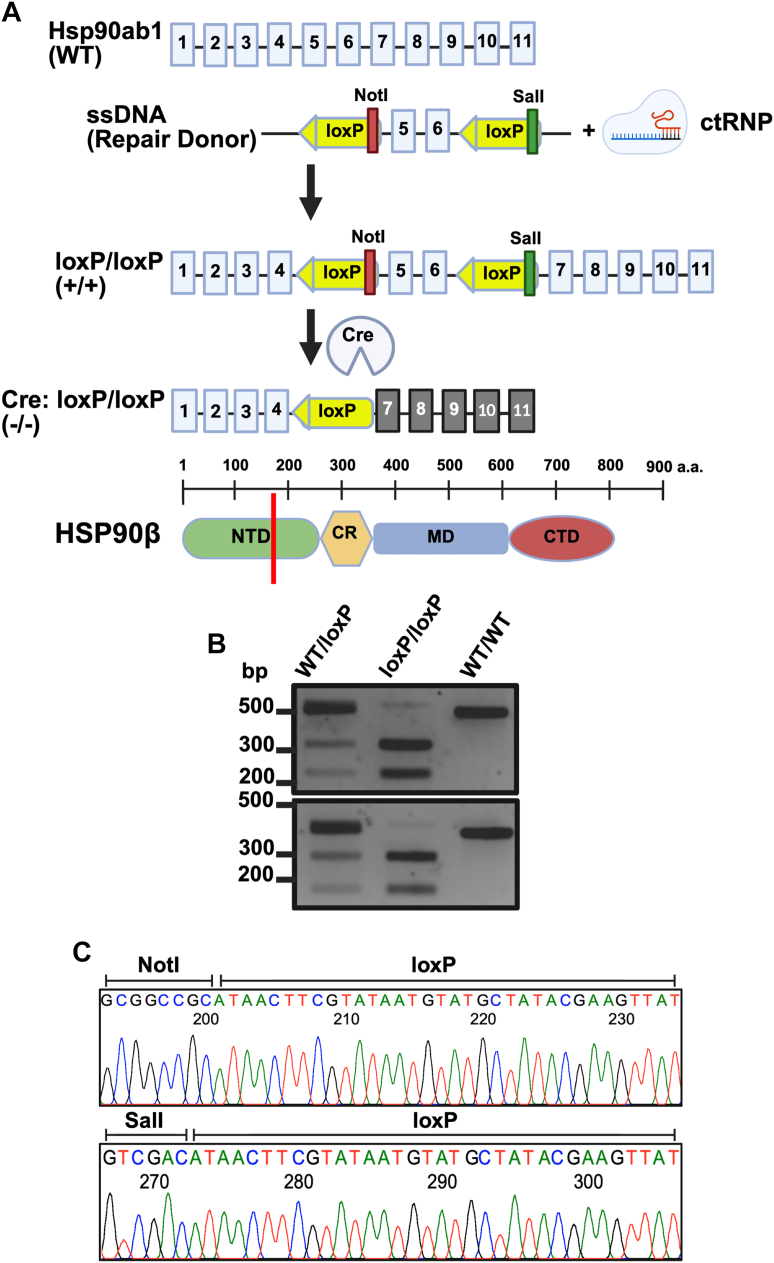
**Design and validation of the Hsp90ab1 floxed allele.***A*, schematic of the Hsp90ab1 genomic locus showing loxP sites flanking exons 5 and 6, with diagnostic NotI and SalI restriction sites incorporated into introns 4 and 6 *via* single-stranded DNA repair donor, respectively (*top*). Cre-mediated excision removes exons 5 and 6, resulting in a frameshift and premature termination (*middle*). The predicted truncated HSP90β protein contains only the N-terminal domain (NTD), with a premature stop codon at amino acid 171 (*red vertical line*) (*bottom*). Domain organization of full-length HSP90β is shown for reference: NTD, N-terminal domain; CR, charged linker; MD, middle domain; CTD, C-terminal domain. *B*, PCR genotyping followed by agarose gel electrophoresis of wildtype (WT/WT), heterozygous (WT/loxP), and homozygous (loxP/loxP) founder mice. Band sizes are indicated in base pairs (bp). *C*, Sanger sequencing of PCR products from a homozygous founder confirms loxP insertion in intron 4 (*top*) and intron 6 (*bottom*), including the adjacent restriction sites used for genotyping. HSP90, heat shock protein 90.

To generate the conditional allele, we used the *Easi*-CRISPR/Cas9 approach to target introns 4 and 6 of the *Hsp90ab1* gene with two guide RNAs and a ssDNA donor template (19, 20). LoxP sites were introduced to flank exons 5 and 6, together with diagnostic NotI and SalI restriction sites to facilitate genotyping (Fig. 1*A*). Correct targeting was confirmed by restriction digestion and Sanger sequencing (Fig. 1, *B* and *C*).

CRISPR/Cas9–mediated integration produced mice homozygous for the floxed *Hsp90ab1* allele (*Hsp90ab1*^*fl/fl*^) (Fig. 1*A*). Founder animals were backcrossed for several generations onto the 129SVE/C57BL/6J mixed background to minimize unintended genetic variation. All mice were free of the *rd1* and *rd8* mutations and were homozygous for the *Rpe65* Leu450 allele. Floxed mice where exons 5 and 6 are flanked by loxP sites (*fl*) were crossed with a *Crx*-Cre driver (12-kb cone-rod homeobox promoter) line to generate *Crx-*Cre*; Hsp90ab1*^*fl/fl*^ (hereafter referred to as *Crx-*Cre: HSP90β^*−/−*^).

The 12-kb *Crx*-Cre transgene initiates recombinase expression at embryonic day 12.5 in cells expressing *Crx* and *Otx2*, including photoreceptors, bipolar cells, and the retinal pigment epithelium (RPE) (22, 23, 24, 25, 26, 27). Cre-mediated excision of exons 5 and 6 introduced a frameshift mutation predicted to generate a truncated HSP90β protein consisting of the N-terminal domain (171 amino acids). This truncated protein lacks both the middle domain required for client binding and the C-terminal dimerization domain essential for HSP90 function (28, 29, 30, 31, 32) (Fig. 1*A*).

**Figure 2:**
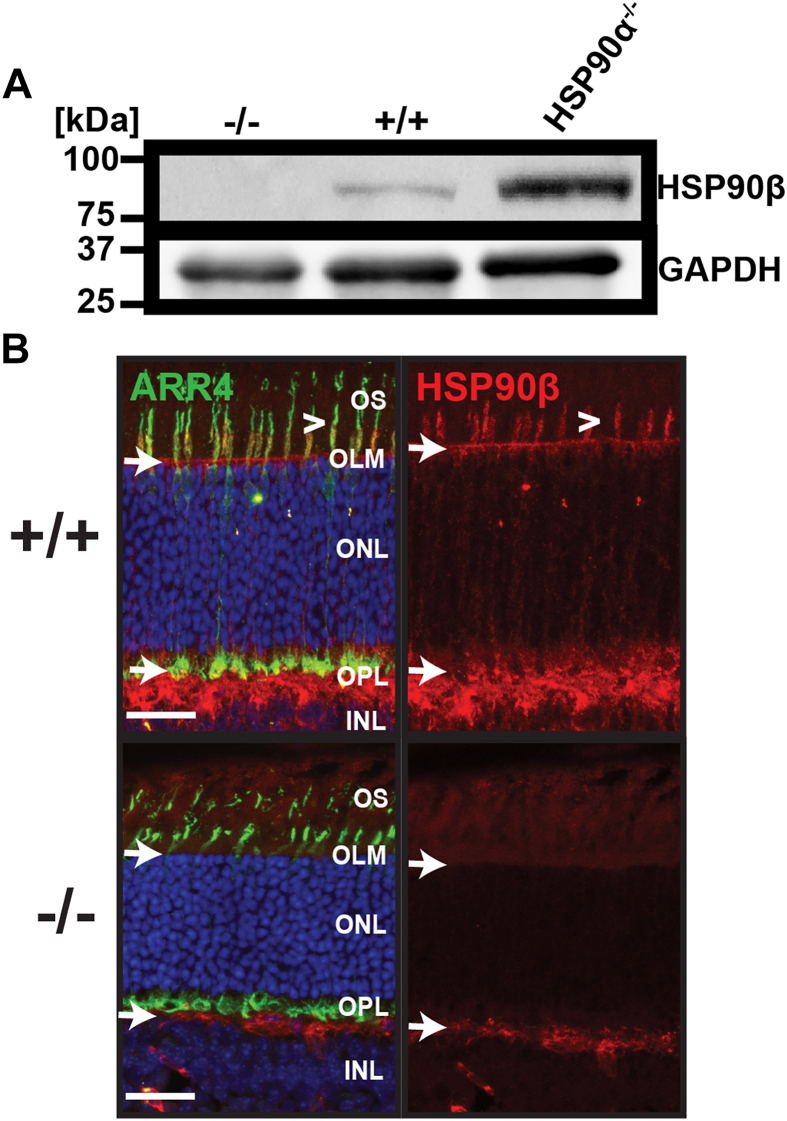
**Validation of Crx-Cre–mediated deletion of HSP90β in the retina.***A*, immunoblot analysis of HSP90β levels in retinal lysates from Crx-Cre: Hsp90ab1fl/fl (^−/−^), floxed control Hsp90ab1fl/fl (^+/+^), and HSP90α knockout (HSP90α^−/−^) mice. GAPDH was used as a loading control. *B*, immunohistochemistry (IHC) of retinal cross sections stained for HSP90β (*red*), DAPI (*blue*), and cone arrestin (ARR4, *green*). The scale bar represents 20 μm. GCL, ganglion cell layer; INL, inner nuclear layer; ONL, outer nuclear layer; OS, outer segments; HSP90, heat shock protein 90; DAP1, death-associated protein 1.

To confirm loss of HSP90β protein, we performed immunoblot analysis using retinal lysates from *Crx-*Cre: HSP90β^*−/−*^ mice and floxed littermate controls lacking Cre (*Hsp90ab1*^*fl/fl*^, hereafter referred to as HSP90β^*+/+*^). Control retinas showed comparable expression levels of HSP90β and HSP90α. In contrast, HSP90β protein was absent in retinas from *Crx-*Cre*:* HSP90β^*−/−*^ mice, while HSP90α expression was unchanged. GAPDH, the housekeeping protein, was used as a loading control (Fig. 2*A*).

To validate the spatial specificity of Cre expression, we first crossed the 12-kb *Crx-*Cre line with a tdTomato reporter mouse. The 12-kb line showed robust reporter activity in photoreceptors but also exhibited ectopic (leaky) expression in the RPE (Fig. S1). To achieve higher spatial fidelity, we utilized a 219-kb BAC-*Crx*-Cre line, which has been shown to more accurately recapitulate endogenous Crx expression (22). Validation with tdTomato reporters confirmed that BAC-*Crx-Cre* expression was restricted to the neural retina with no detectable recombination in the RPE (Fig. S1). While phenotypic analyses of *Hsp90ab1* deletion yielded concordant results with both drivers, the BAC-*Crx-Cre* line was employed for all subsequent experiments to ensure retina-specific ablation unless indicated otherwise.

### HSP90β is dispensable for cone development, photoreceptor function, and protein homeostasis

Global deletion of HSP90β results in embryonic lethality and early developmental defects, demonstrating its essential role during development (18). Consistent with this finding, single-cell RNA sequencing studies show that HSP90β is highly expressed in retinal progenitor cells during early retinal development, with expression declining over time. Immunoblot analyses further demonstrate robust retinal HSP90β expression during early postnatal development, followed by a marked reduction after postnatal day 14 (14). In the adult retina, we detected HSP90β expression predominantly in cone inner segments and in the inner retina (9). Together, these observations indicate that HSP90β is expressed during early retinal development and is detectable in cones at later stages.

To determine whether cone development or maintenance depends on HSP90β, we performed immunostaining on postnatal day 30 (P30) retinal sections from *Crx*-Cre; *Hsp90ab1*^*fl/fl*^ mice (*Crx*-Cre: HSP90β^−/−^) and floxed littermate controls (*Hsp90ab1*^*fl/fl*^ or HSP90β^+/+^). In HSP90β^+/+^ retinas, HSP90β colocalized with cone arrestin (ARR4) in the inner segments of cone photoreceptors (Fig. 2*B*, arrowhead). HSP90β immunoreactivity was also detected at the outer limiting membrane (Fig. 2*B*, arrow), within the plexiform layers, and in downstream retinal neurons, including bipolar and ganglion cells. In *Crx*-Cre: HSP90β^−/−^ retinas, HSP90β staining was absent from photoreceptors and the outer limiting membrane and was reduced in downstream retinal neurons (Fig. 2*B*).

Despite loss of HSP90β expression in photoreceptors, overall retinal lamination was preserved, with normal organization of nuclear layers throughout the retina. Cone and rod morphology appeared normal, and no differences were observed in the localization of ARR4 or phosphodiesterase 6 β (PDE6β) between HSP90β^+/+^ and *Crx*-Cre: HSP90β^−/−^ retinas (Fig. S2). BAC-*Crx*-Cre: HSP90β^−/−^ mice showed similar preservation of retinal architecture, and retinal flat mounts showed no differences in cone density (Fig. S3).

Photoreceptor function was evaluated in *Crx*-Cre: HSP90β^−/−^ mice and floxed littermate controls (HSP90β^+/+^) using electroretinography (ERG). ERG recordings consist of distinct waveform components generated by different retinal cell types. The a-wave, a negative deflection, reflects photoreceptor activity, whereas the b-wave, a positive deflection, primarily arises from bipolar cells.

**Figure 3:**
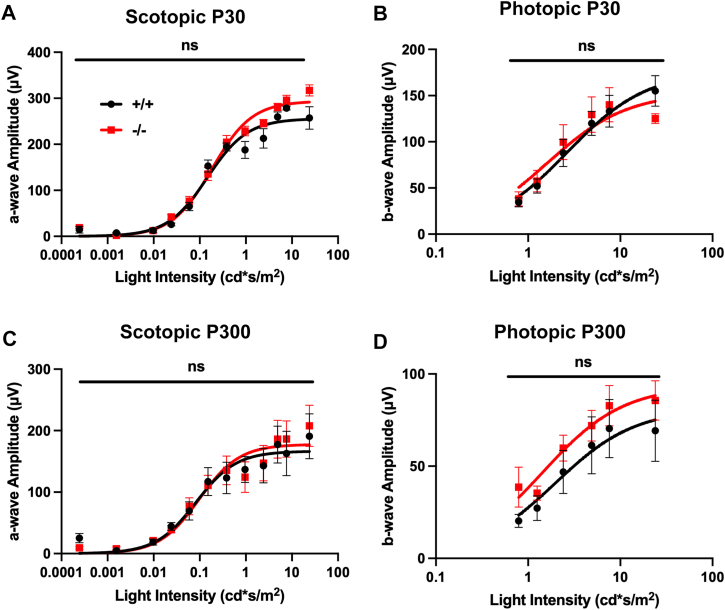
**HSP90β is not essential for photoreceptor function.***A*, scotopic a-wave sensitivity curves from HSP90β+/+ (*black*, +/+) and Crx-Cre: HSP90β^−/−^ (*red*, ^−/−^) mice at postnatal day 30 (P30); n = 4. *B*, photopic b-wave sensitivity curves from the same genotypes at P30; n = 4. *C*, scotopic a-wave sensitivity curves from HSP90β^+/+^ (*black*, ^+/+^) and Crx-Cre: HSP90β^−/−^ (*red*, ^−/−^) mice at postnatal day 300 (P300); n = 4. *D*, photopic b-wave sensitivity curves from the same genotypes at P300; n = 4. Error bars represent mean ± SEM. ns, not significant (*p* > 0.05). HSP90, heat shock protein 90; SEM, scanning electron microscope.

From P30 through P300, *Crx*-Cre: HSP90β^−/−^ mice exhibited scotopic (dark-adapted) a-wave amplitudes comparable to those of control mice, indicating preserved rod photoreceptor function (Fig. 3, *A* and *C*). Photopic (light-adapted) ERG recordings assessing cone-driven responses likewise showed no differences between *Crx*-Cre: HSP90β^−/−^ mice and controls at any time point examined (Fig. 3, *B* and *D*). BAC-*Crx*-Cre: HSP90β^−/−^ mice similarly showed no differences in scotopic or photopic ERG responses at ages greater than 2 months (Fig. S4).

**Figure 4:**
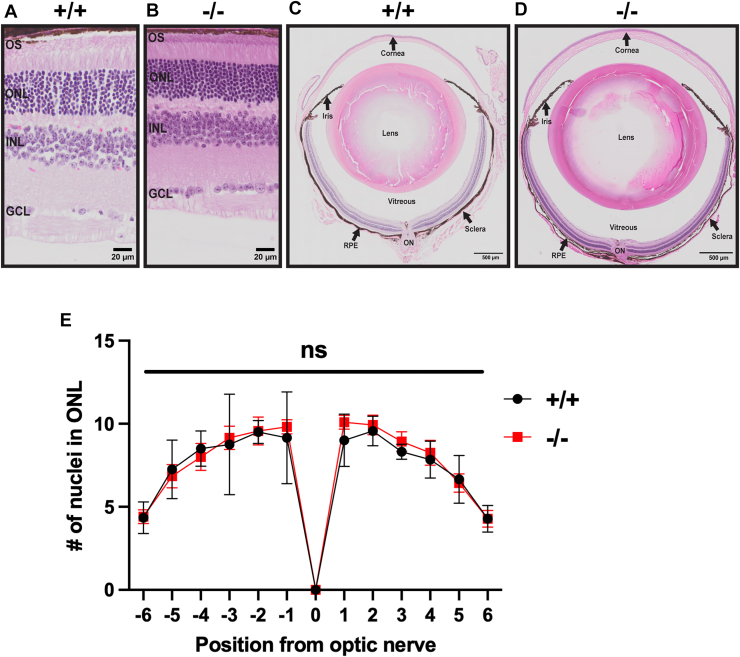
**Photoreceptor nuclei are preserved in the absence of HSP90β at advanced age.***A* and *B*, light microscopy of hematoxylin and eosin (H&E) stained retinal cross-sections from (*A*) HSP90β+/+ (+/+) and (*B*) Crx-Cre: HSP90β−/− (−/−) mice at postnatal day 480 (P480). *C* and *D*, low magnification H&E-stained whole-eye cross sections from (*C*) HSP90β+/+ (+/+) and (*D*) Crx-Cre: HSP90β−/− (−/−) mice at P480. *E*, quantification of outer nuclear layer (ONL) nuclei across the retina at P480 of HSP90β+/+ (+/+) and Crx-Cre: HSP90β−/− (−/−). Nuclei were counted in triplicate at six equally spaced locations on both the superior and inferior sides of the optic nerve head. Data are presented as spider plots. Two-way ANOVA; ns, not significant (*p* > 0.05); n = 3 biological replicates with 3 technical replicates per location. The scale bar represents 20 μm. GCL, ganglion cell layer; HSP90, heat shock protein 90; INL, inner nuclear layer; ON; optic nerve; ONL, outer nuclear layer; OS, outer segments.

**Figure 5:**
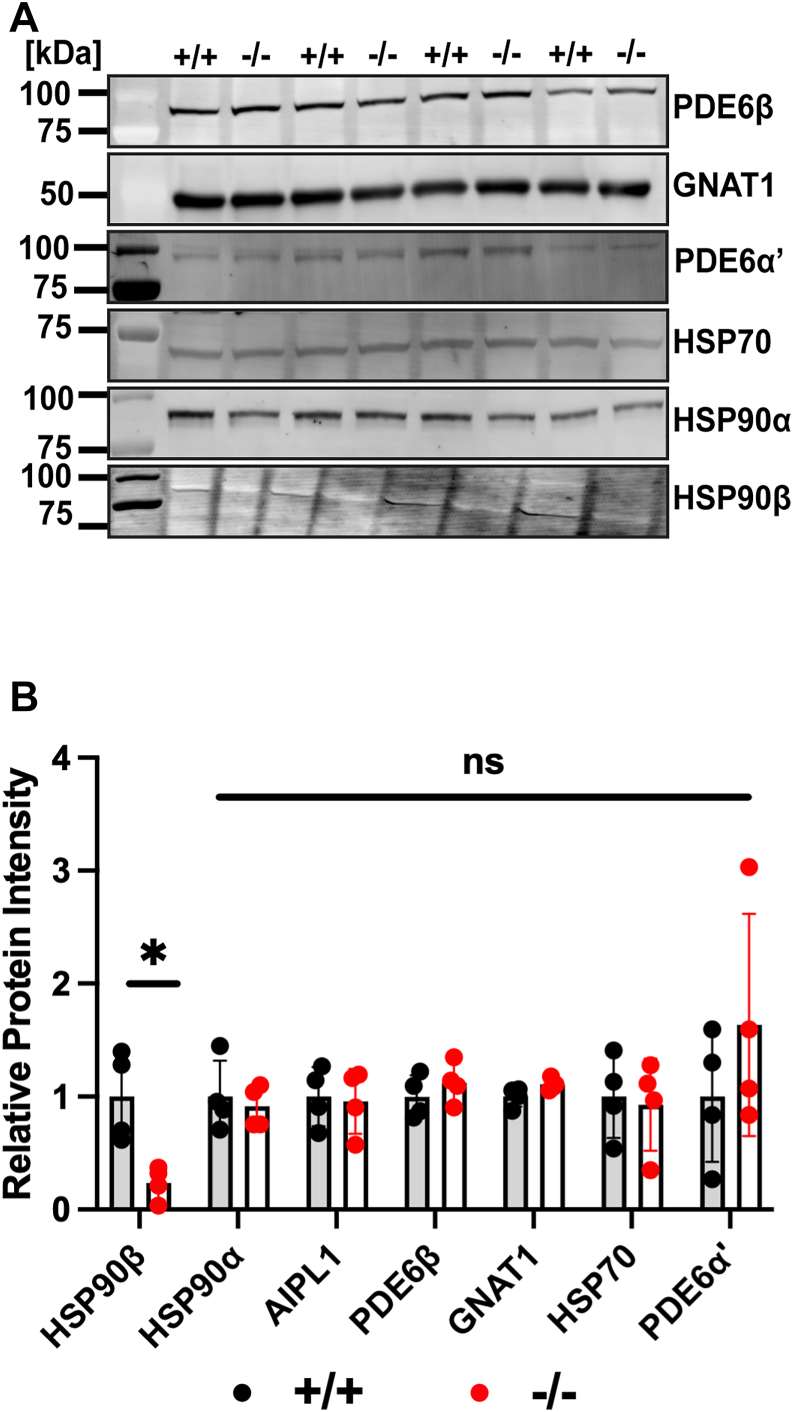
**HSPs and phototransduction protein levels are unchanged in the absence of HSP90β.***A*, immunoblot analysis of PDE6β, GNAT1, PDE6α′, HSP70, HSP90α and HSP90β in retinal lysates from HSP90β+/+ (+/+) and Crx-Cre: HSP90β−/− (−/−) mice at postnatal day 300 (P300). Total protein staining was used as a loading control. *B*, quantification of protein levels from (*A*) in HSP90β+/+ (*black*, +/+) and Crx-Cre: HSP90β−/− (*red*, −/−) mice; n = 4. Data represent mean ± SD. ns, not significant (*p* > 0.05); ∗*p* = 0.02. HSP, heat shock protein; PDE6, phosphodiesterase 6.

Retinal architecture and photoreceptor survival were assessed in aged animals to determine whether loss of HSP90β affects long-term photoreceptor maintenance. Hematoxylin and eosin (H&E) staining of retinal cross-sections from *Crx*-Cre: HSP90β^−/−^ and control mice at postnatal day 480 revealed preserved retinal organization (Fig. 4, *A*–*D*). Outer nuclear layer thickness and photoreceptor nuclear counts were comparable between genotypes, indicating no detectable loss of photoreceptors with age (Fig. 4*E*). Immunoblot analyses using P300 retinal lysates showed similar levels of HSP90α, PDE6β, and HSP70 between genotypes, with selective loss of HSP90β in KO samples (Fig. 5).

Collectively, loss of HSP90β alone does not disrupt cone development, photoreceptor function, localization of selected phototransduction proteins, or long-term retinal maintenance under the conditions examined.

### Combined loss of HSP90 paralogs abolished cone photoreceptor function

Our studies show that global loss of HSP90α leads to rod degeneration, but cones are spared. Consistent with this observation, removal of HSP90α in cone-enriched retina (*Nrl* ^−/−^) showed normal function at the latest age tested at 6 months (9). In the current study, we find that HSP90β is dispensable for photoreceptor development and maintenance. Together, these findings suggest that the cytosolic paralogs HSP90α and HSP90β may compensate for each other in cones.

**Figure 6:**
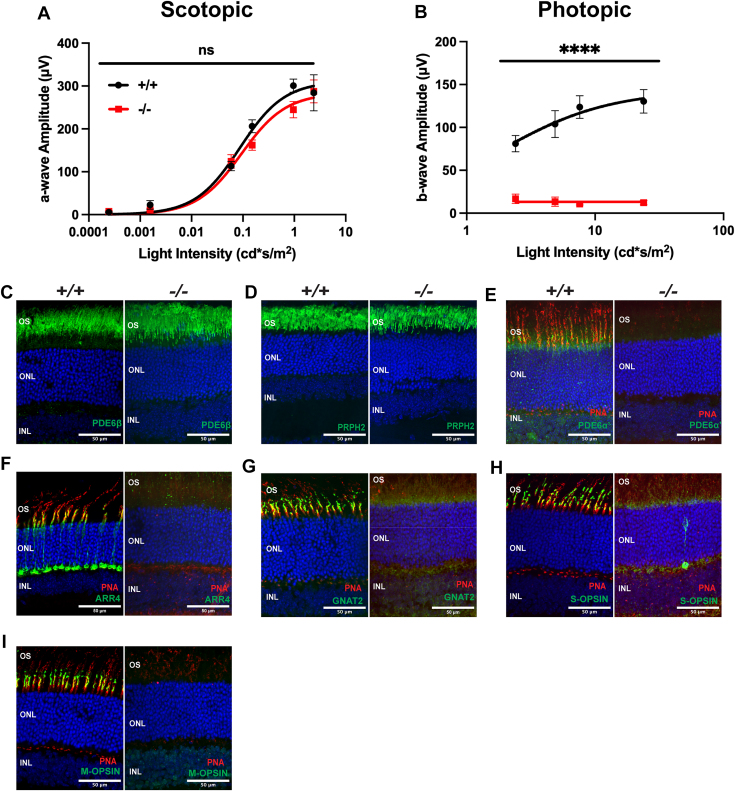
**Cytosolic HSP90 paralogs compensate for each other in cones.***A*, scotopic a-wave sensitivity curves from HSP90+/+ control (*black*, +/+) and Cone-Cre: HSP90−/− (*red*, −/−) mice at P30-45. *B*, photopic b-wave sensitivity curves from the same genotypes at P30-45; n = 3. Data represent mean ± SEM. ns, not significant (*p* > 0.05); ∗∗∗∗*p* < 0.0001. *C*, *D*, *E*, *F*, *G*, *H*, and *I*, immunohistochemistry of retinal cross sections from HSP90+/+ (+/+) and Cone-Cre: HSP90−/− (−/−) mice at postnatal days 30 to 45 following ERG experiments. *C*, PDE6β (*green*) and DAPI (*blue*). *D*, PRPH2 (*green*) and DAPI (*blue*). *E*, PDE6α′ (*green*), PNA (*red*), and DAPI (*blue*). *F*, cone arrestin (ARR4, *green*), PNA (*red*), and DAPI (*blue*). *G*, cone transducin (GNAT2, *green*), PNA (*red*), and DAPI (*blue*). *H*, S-opsin (*green*), PNA (*red*), and DAPI (*blue*). *I*, M-opsin (*green*), PNA (*red*), and DAPI (*blue*). The scale bar represents 50 μm. DAP1, death-associated protein 1; ERG, electroretinography; HSP, heat shock protein; PDE6, phosphodiesterase 6; PNA, peanut agglutin; SEM, scanning electron microscope.

To test this possibility, we crossed *Hsp90ab1*^*fl/fl*^ mice with *HRGP*-Cre driver (Cone-Cre) line to generate *HRGP-*Cre*; Hsp90ab1* ^*fl/fl*^, which was then crossed with HSP90α^−/−^ to generate Cone-Cre: HSP90^−/−^ animals. In this model, Cre recombinase is specifically expressed in cones starting at P10, with maximal expression observed at P30 (21). When we tested photoreceptor function by ERG at P35-45, rod function was preserved, but we noted a complete loss of photopic (cone) vision in Cone-Cre: HSP90^−/−^ (Fig. 6, *A* and *B*). In contrast, the littermate controls lacking Cre recombinase exhibited normal cone responses (Fig. 6, *A* and *B*).

Immunohistochemistry confirmed proper localization of rod markers, including PDE6β and PRPH2 (Fig. 6, *C* and *D*). In contrast, at P30-45, cone markers, including cone PDE6α’ (PDE6α′), cone arrestin (ARR4), cone transducin α subunit (GNAT2), S-opsin, M-opsin, and peanut agglutinin, were absent, sparse, or mislocalized, consistent with rapid degeneration of cone photoreceptors in the absence of cytosolic HSP90 paralogs (Fig. 6, *E*–*I*).

These findings demonstrate the essential role of cytosolic HSP90 in cone photoreceptor function and survival.

### Combined loss of cytosolic HSP90 paralogs abolishes photoreceptor function

Previous work from our laboratory demonstrated that whole-body deletion of HSP90α did not affect photoreceptor development or visual function through P45. However, we observed progressive rod degeneration and loss of rod function at later stages, despite upregulation of HSP90β (9). Loss of HSP90β alone did not produce detectable retinal phenotypes, motivating examination of the consequence of simultaneously removing both cytosolic HSP90 paralogs from the retina.

To generate a retinal double KO, *Crx*-Cre: HSP90β^−/−^ mice were crossed with heterozygous HSP90α^+/−^ mice to obtain *Crx-*Cre*:* HSP90^*−/−*^ animals. These mice exhibited a complete loss of ERG response by P30 under photopic and scotopic conditions (Fig. S5, *A*–*D*). Histological analysis at P9 revealed severely disrupted retinal morphology with whorls in the outer nuclear layer in double KOs (Fig. S5, *F* and *H*) compared to normal retinal architecture in control retinas (Fig. S5, *E* and *G*). *Crx-*Cre*:* HSP90^*−/−*^ mice at P21 also exhibited systemic abnormalities, including malocclusion and reduced body size (Fig. S5, *I* and *J*).

Together, simultaneous loss of HSP90α and HSP90β using the 12-kb *Crx*-Cre driver causes early and severe retinal disruption, leading to complete loss of photoreceptor function and growth defects, including malocclusion.

### Retina-restricted loss of cytosolic HSP90 is sufficient to eliminate photoreceptor function

Because the 12-kb *Crx-*Cre driver mediates recombination in tissues outside the neural retina, including the RPE and regions of the brain, it produced confounding pleiotropic effects, such as malocclusion and stunted postnatal growth. To determine whether loss of cytosolic HSP90β in the retina alone is sufficient to drive photoreceptor dysfunction, we used the BAC-*Crx-*Cre driver. Validation with tdTomato reporters confirmed that Cre expression in this line is restricted to the neural retina, thereby isolating the retinal phenotype from systemic developmental defects (Fig. S1) (22).

**Figure 7:**
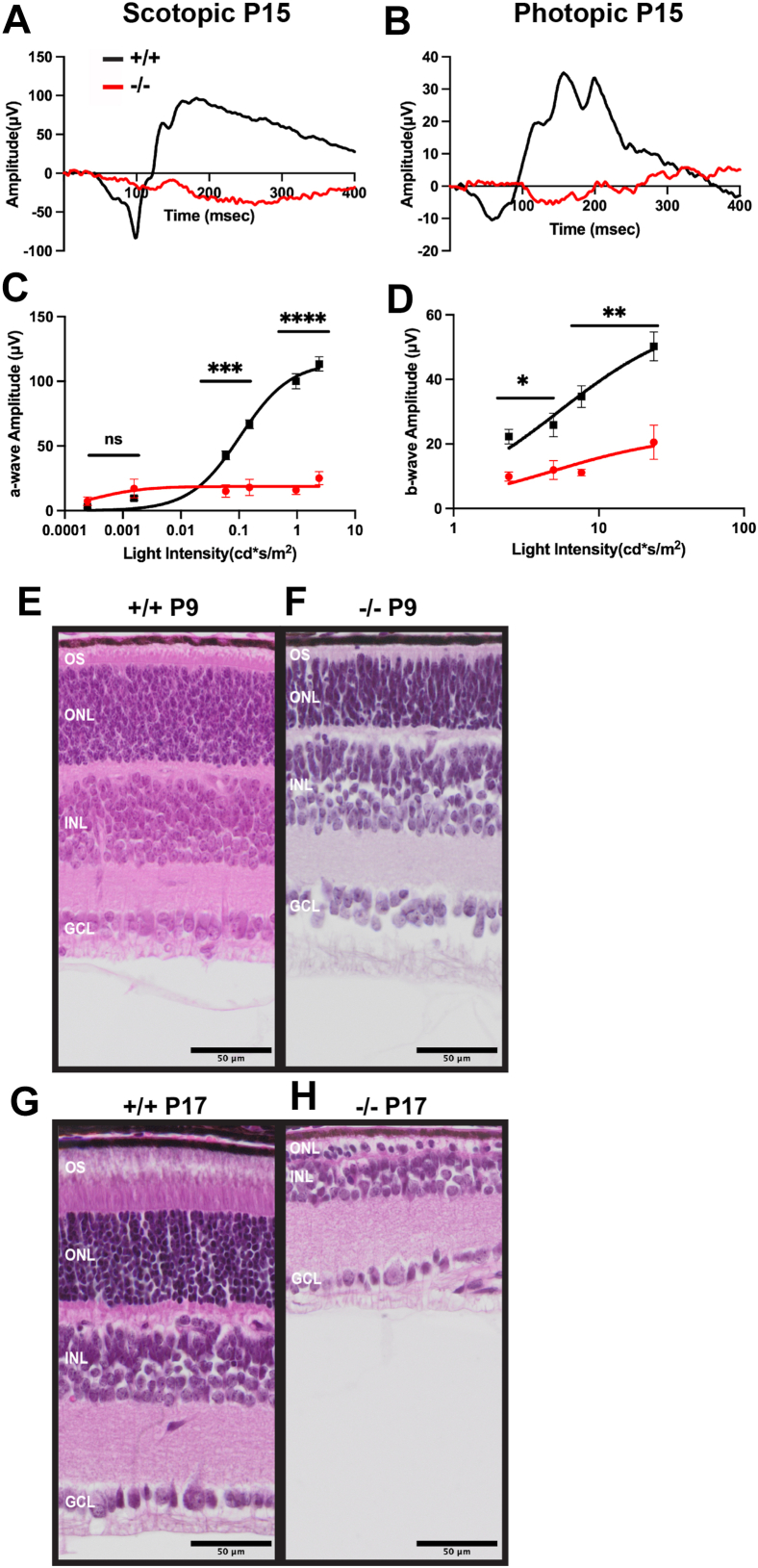
**Retina-restricted loss of cytosolic HSP90 abolishes photoreceptor function and causes rapid degeneration.***A*, representative scotopic ERG responses from HSP90+/+ control (*black*, ^+/+^) and BAC-Crx-Cre: HSP90^−/−^ (*red*, ^−/−^) postnatal day 15 (P15) recorded at −0.1 log cd∗s/m2. *B*, representative photopic ERG responses from HSP90^+/+^ control (*black*, ^+/+^) and BAC-Crx-Cre: HSP90^−/−^ (*red*, ^−/−^) mice recorded at 4.8 log cd∗s/m2. *C*, scotopic a-wave sensitivity curves from HSP90^+/+^ control (*black*, ^+/+^) and BAC-Crx-Cre: HSP90^−/−^ (*red*, ^−/−^) mice at P15. *D*, photopic b-wave sensitivity curves from the same genotypes at P15. Data represent mean ± SEM. ns, not significant (*p* > 0.05); ∗*p* < 0.05; ∗∗∗*p* < 0.001; ∗∗∗∗*p* < 0.0001. *E* and *F*, H&E stained retinal cross-sections from HSP90^+/+^ control (^+/+^) (*E*) and BAC-Crx-Cre: HSP90^−/−^ (^−/−^) (*F*) at postnatal day 9 (P9). The scale bar represents 50 μm. *G* and *H*, H&E-stained retinal cross-section from HSP90^+/+^ control (^+/+^) (*G*) and BAC-Crx-Cre: HSP90^−/−^ (^−/−^) (*H*) at postnatal day 17 (P17). The scale bar represents 50 μm. BAC, bacterial artificial chromosome; H&E, hematoxylin and eosin; HSP90, HSP, heat shock protein 90.

After several rounds of breeding, we generated a BAC*-Crx*-Cre: *Hsp90aa1*^*−/−*^*: Hsp90ab1*^*fl/fl*^ (referred to as BAC*-Crx*-Cre: HSP90^−/−^). These animals did not exhibit systemic abnormalities and showed normal growth and ocular morphology. BAC*-Crx*-Cre: HSP90^−/−^ exhibit significantly reduced photoreceptor function as early as P15, with reductions in both scotopic and photopic responses (Fig. 7, *A*–*D*).

These findings demonstrate that retina-restricted loss of cytosolic HSP90 paralogs leads to significant loss of rod and cone photoreceptor function very early.

### Cytosolic HSP90 is required for photoreceptor OS maturation

We hypothesized, based on the total loss of photoreceptor function as early as P15, that HSP90 is needed for normal photoreceptor development. To test this hypothesis, we performed H&E staining and examined retinal morphology in BAC*-Crx*-Cre: HSP90^−/−^ mice at P9 using light microscopy. Retinal lamination appeared grossly normal, although abnormalities were evident in the OSs as compared to controls (Fig. 7, *E* and *F*). Retinal morphology was also examined at P17 following ERG recordings. Normal OS development was observed in controls, whereas BAC*-Crx*-Cre: HSP90^−/−^ mice exhibited extensive loss of photoreceptor nuclei (Fig. 7, *G* and *H*).

**Figure 8:**
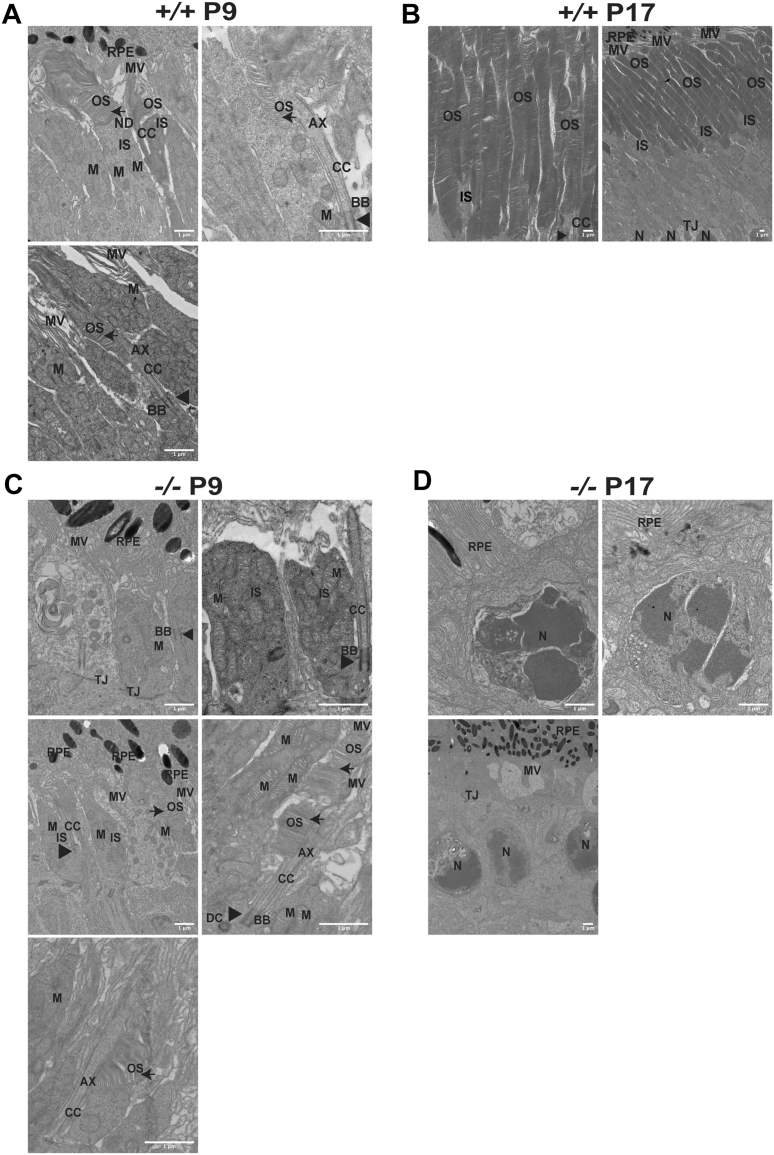
**Photoreceptor outer segment ultrastructure fails to develop in retina lacking cytosolic HSP90.** Transmission electron microscopy (TEM) of retinal cross sections from HSP90^+/+^(^+/+^) (*A* and *B*) and BAC-Crx-Cre: HSP90^−/−^ (^−/−^) (*C* and *D*) mice at postnatal day 9 (P9) (*A* and *C*) and postnatal day 17 (P17) (*B* and *D*); n = 3 per group. *Arrows* indicate photoreceptor outer segments, arrowheads indicate basal bodies. the scale bar represents 1 μm. AX, axoneme; BAC, bacterial artificial chromosome; CC, connecting cilium; DC, daughter centriole; HSP90, HSP, heat shock protein 90; M, mitochondria; MV, microvilli; N, nucleus; ONL, outer nuclear layer; OS, outer segments; RPE, retinal pigment epithelium; TJ, tight junction.

We then performed transmission electron microscopy (TEM) imaging of samples collected from P9 and P17 BAC*-Crx*-Cre: HSP90^−/−^ mice to provide a more detailed view of OS development (Fig. 8). We observe normal OS development in P9 control mice with the properly formed basal body and connecting cilium, and nascent disc structures (Fig. 8*A*), which matured into well-organized OSs by P17 (Fig. 8*B*). In contrast, the BAC*-Crx*-Cre: HSP90^−/−^ photoreceptors retained basal body and connecting cilium at P9, but majority of photoreceptors fail to form OSs (Fig. 8*C*). By P17, the retina was severely degenerated, with the loss of outer and inner segments and only a few rows of the outer nuclear layer remaining (Fig. 8*D*).

These observations indicate that cytosolic HSP90 is not required for the initial formation of photoreceptors but is necessary for subsequent OS development and photoreceptor survival.

### OS protein localization is disrupted in the absence of cytosolic HSP90

**Figure 9:**
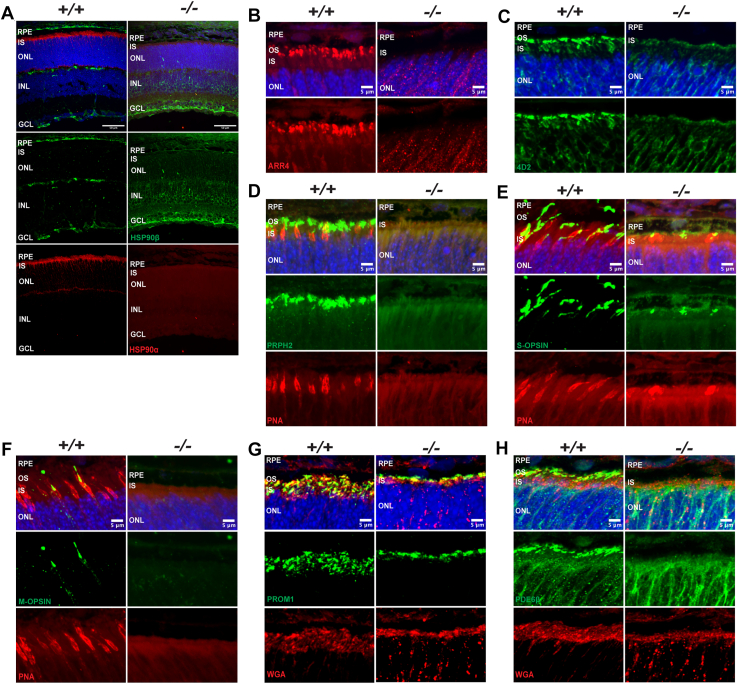
**Outer segment protein accumulation is impaired in the absence of cytosolic HSP90.** Immunohistochemical (IHC) staining of retinal cross sections from HSP90^+/+^ (^+/+^) and BAC-Crx-Cre: HSP90^−/−^ (^−/−^) mice at postnatal day 9 (P9). *A*, Ηeat Shock Protein 90 β (HSP90β, *green*) and Heat Shock Protein 90 α (HSP90α, *red*). *B*, cone arrestin (ARR4, red) and DAPI (*blue*). *C*, rhodopsin (4D2, *green*) and DAPI (*blue*). *D*, peripherin-2 (PRPH2, *green*), peanut agglutinin (PNA, *red*), and DAPI (*blue*). *E*, S-opsin (*green*), peanut agglutinin (PNA, *red*), and DAPI (*blue*). *F*, M-opsin (*green*), peanut agglutinin (PNA, *red*), and DAPI (*blue*). *G*, Prominin-1 (PROM1, *green*), Wheat Germ agglutinin (WGA, *red*), and DAPI (*blue*). *H*, rod phosphodiesterase 6β (PDE6β, *green*), Wheat Germ Agglutinin (WGA, *red*), and DAPI (*blue*). The scale bars represent 50 μm (*A*) and 5 μm (*B*, *C*, *D*, *E*, *F*, *G*, and *H*,). GCL, ganglion cell layer; INL, inner nuclear layer; ONL, outer nuclear layer; OS, outer segments. BAC, bacterial artificial chromosome; DAP1, death-associated protein 1; HSP, heat shock protein 90.

To determine whether the OS defects reflected impaired protein localization, we performed immunohistochemistry on retinal cryosections using antibodies against rod and cone OS proteins (Fig. 9). Loss of HSP90α and HSP90β in photoreceptors was confirmed (Fig. 9*A*), although HSP90β remained detectable in inner retinal layers, consistent with the specificity of the *BAC-Crx* Cre mouse line and compensatory upregulation in the absence of HSP90α (9).

Rhodopsin localized to the OSs in control retinas but was highly reduced in the presumptive OS layer in BAC*-Crx*-Cre: HSP90^−/−^ retinas (Fig. 9*C*). Cone markers, including peanut agglutinin, ARR4, S-opsin, and M-opsin, showed robust staining in controls but were absent, sparse, or mislocalized in BAC*-Crx*-Cre: HSP90^−/−^ retinas (Fig. 9, *B*, *E* and *F*).

Given these defects, we examined proteins involved in OS morphogenesis, including peripherin-2 (PRPH2) and prominin-1 (33, 34, 35, 36, 37, 38, 39). Both proteins showed normal localization in control retinas, whereas PRPH2 was absent and prominin was properly localized in BAC*-Crx*-Cre: HSP90^−/−^ mice (Fig. 9, *D* and *G*).

Together, these findings indicate that loss of cytosolic HSP90 disrupts the localization of key OS proteins, indicating a failure of OS maturation.

### Loss of cytosolic HSP90 paralogs leads to reduced expression of peripherin

**Figure 10:**
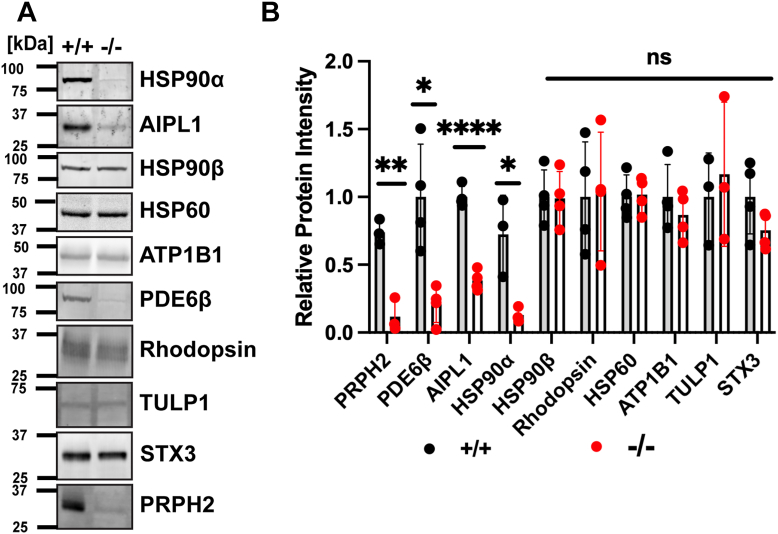
**Loss of cytosolic HSP90 leads to a marked reduction in peripherin.***A* and *B*, immunoblot analysis of HSP90α (*p* = 0.03), AIPL1 (*p* = 0.00003), HSP90β, HSP60, Na-K ATPase β1 (ATP1B1), PDE6β (*p* = 0.02), Rhodopsin, TUB-Like Protein 1 (ULP1), Syntaxin (STX3), and Peripherin-2 (PRPH2) (*p* = 0.003) in retinal lysates from HSP90^+/+^ (^+/+^) and Crx-Cre: HSP90^−/−^ (^−/−^) mice at postnatal day 9 (P9). Total protein staining was used as a loading control. *B*, quantification of protein levels from (*A*) in HSP90^+/+^ (*black*, ^+/+^) and Crx-Cre: HSP90^−/−^ (*red*, ^−/−^) mice; n = 3 to 4. Data represent mean ± S.D. ns, not significant (*p* > 0.05); ∗*p* < 0.05; ∗∗*p* < 0.01; ∗∗∗∗*p* < 0.0001. HSP, heat shock protein 90; PDE6β, phosphodiesterase 6 β.

We performed immunoblotting on retinal extracts from P9 BAC-*Crx*-Cre: HSP90^−/−^ mice and littermate controls to assess changes in protein levels, focusing on OS proteins and known HSP90 clients. Consistent with enrichment of HSP90α in photoreceptors (Fig. 9*A*), its levels were markedly reduced in retinal lysates from the KO animals (Fig. 10). In contrast, HSP90β is expressed in the inner retina, and loss of HSP90α leads to compensatory upregulation of HSP90β (9); accordingly, no measurable change in total HSP90β levels were detected (Fig. 10). However, immunohistochemistry revealed a specific loss of HSP90β in the outer retina, validating the model (Fig. 9*A*).

We observed a significant reduction in rod PDE6 and AIPL1 levels compared with control mice, consistent with a role for HSP90 in regulating PDE6 abundance (40, 41, 42) (Fig. 10). In contrast, rhodopsin levels were unchanged, and inner segment markers, including Na^+^/K^+^-ATPase β1 subunit and Tubby-like protein 1, were preserved (Fig. 10). These results support a role for HSP90 as a chaperone for AIPL1 and PDE6 biosynthesis (40, 41, 42).

We also observed a marked reduction in PRPH2, consistent with immunohistochemical findings (Figs. 9*D* and 10). PRPH2 is a structural protein required for photoreceptor disc enclosure and flattening, and its loss leads to failure of OS development and rapid degeneration (9, 35, 43).

Together, these findings indicate that cytosolic HSP90 is required for the stability of key OS proteins and proper OS development in photoreceptors.

## Discussion

This study defines stage- and cell type–specific functions of cytosolic HSP90 paralogs in retinal development and photoreceptor function using conditional mouse models lacking HSP90β alone or both cytosolic paralogs. Conditional alleles were designed to eliminate all functional domains of the targeted proteins, enabling assessment of paralog-specific and shared requirements for cytosolic HSP90 activity in the retina.

Loss of HSP90β alone did not disrupt retinal development, photoreceptor survival, or visual function, despite enrichment of HSP90β in cone inner segments. These findings were consistent across two independent Cre driver lines and extended through advanced ages. In contrast, HSP90α deficiency leads to progressive rod degeneration, sparing the cones (9). Together, these results indicate that neither cytosolic HSP90 paralog is individually required for early retinal development or cone maintenance.

In contrast, simultaneous removal of both cytosolic HSP90 paralogs resulted in severe retinal phenotypes. Deletion of HSP90α and HSP90β using the 12-kb *Crx*-Cre driver caused marked disruption of retinal organization and complete loss of photoreceptor function. In addition, a retina-restricted BAC-*Crx*-Cre was used, which reproduced the complete loss of photoreceptor function without systemic abnormalities, establishing that cytosolic HSP90 activity is required for photoreceptor viability and function.

A requirement for cytosolic HSP90 activity was also evident in cones when both paralogs were removed selectively from this cell type. Cone-specific deletion of HSP90α and HSP90β using an HRGP-Cre driver resulted in rapid loss of photopic responses and degeneration of cone photoreceptors over a short postnatal interval. In this model, Cre recombinase activity initiates after P10. It reaches maximal levels by approximately P30 (21). Together with findings from the BAC*-Crx*-Cre HSP90 model, our results indicate that cytosolic HSP90 activity is required for the maintenance of mature cones. The absence of cone dysfunction following loss of either paralog alone, together with the rapid functional collapse observed after combined deletion, suggests that HSP90α and HSP90β can compensate for one another in cones.

Analysis of retina-restricted double KO mice showed that loss of cytosolic HSP90 does not prevent initial photoreceptor development but interferes with subsequent maturation. Nuclear layers were established by P9, yet photoreceptors rapidly degenerated, with extensive nuclear loss by P17. Structural analyses revealed a pronounced defect in OS formation. Rod and cone OS markers failed to localize appropriately, indicating impaired OS maturation rather than secondary degeneration. Ultrastructural analysis confirmed that while basal bodies and connecting cilia were present, disc morphogenesis is profoundly impaired. These findings place the requirement for cytosolic HSP90 for the elaboration of the OS, identifying a specific developmental step that depends on cytosolic HSP90 activity.

OS disc morphogenesis requires coordinated membrane remodeling and stabilization (44, 45). PRPH2, a tetraspanin protein localized to OS disc rims, is essential for disc formation and photoreceptor maintenance (33, 34, 35, 36). In retina-restricted HSP90 double KO mice, PRPH2 was absent, and OSs failed to form, producing a phenotype resembling that observed in *Prph2* KO models. The loss of PRPH2 suggests that cytosolic HSP90 activity may be required for the stability or maturation of PRPH2. Given the intrinsically disordered nature of the PRPH2 C-terminal domain and the role of HSP90 in stabilizing conformationally labile intrinsically disordered proteins, loss of cytosolic HSP90 activity may render PRPH2 unstable or susceptible to degradation (46, 47, 48). Although a direct interaction between HSP90 and PRPH2 was not examined, the convergence of phenotypes supports a functional link between cytosolic HSP90 activity and disc morphogenesis.

### Limitations

Interpretation of phenotypes generated using the 12-kb *Crx*-Cre driver requires caution due to documented recombination outside the neural retina, including the RPE and other tissues. In this context, systemic defects such as impaired growth and premature lethality likely reflect loss of cytosolic HSP90 activity in nonretinal tissues and complicate attribution of retinal phenotypes to photoreceptor-intrinsic mechanisms. These limitations were addressed by using a retina-restricted BAC-*Crx*-Cre driver, which eliminated systemic abnormalities while preserving the retinal phenotype.

In addition, loss of both cytosolic HSP90 paralogs resulted in rapid and severe photoreceptor degeneration. While this phenotype enabled unambiguous identification of an essential requirement for cytosolic HSP90 activity, the speed and severity of degeneration limit the ability to resolve individual downstream pathways contributing to photoreceptor failure. Given HSP90's broad client repertoire, multiple processes, including OS morphogenesis and general proteostasis stability, are likely affected.

## Conclusions

This study defines essential and overlapping roles for cytosolic HSP90 paralogs in photoreceptor development and function. Although loss of either HSP90α or HSP90β alone is tolerated during retinal development and photoreceptor maintenance, combined loss of cytosolic HSP90 activity disrupts OS maturation, compromises photoreceptor viability, and abolishes visual function.

Cone-specific deletion of both paralogs demonstrates that cytosolic HSP90 activity is required for cone photoreceptor function and survival after development. The preservation of cone structure and function following loss of either paralog alone indicates functional compensation between HSP90α and HSP90β in cones. In contrast, the rapid loss of photopic responses following combined deletion establishes an essential requirement for cytosolic HSP90 activity in mature cones.

Together, these findings show that paralog redundancy buffers the loss of individual HSP90 paralog during photoreceptor development and maintenance, but that cytosolic HSP90 activity is indispensable for OS maturation and sustained photoreceptor function. This work identifies cytosolic HSP90 as a key component of the proteostasis machinery supporting photoreceptor structure and function.

## Experimental procedures

### Design of animal models

*Hsp90aa1* KO mice were generated as previously described (9). Conditional *Hsp90ab1* KO mice were generated using *Easi*-CRISPR/Cas9 technology at the Transgenic Core Laboratory (Johns Hopkins University). Guide RNAs with high predicted cutting efficiency and minimal off-target activity were identified using online CRISPR design tools. Synthetic single-stranded guide RNAs were obtained from Integrated DNA Technologies.

Two guide RNAs (5′-TTAAGAACTCACCCAAGTGC-3′) for intron 4 and (5′-TACTGCTGAACCCTATGAAT-3′) for intron 6 regions flanking exons 5 and 6 of *Hsp90ab1* (ENSG00000096384), were used together with a single-stranded donor repair template (1273 bp). The donor template contained ∼80-bp homology arms corresponding to the guide RNA target regions in introns 4 and 6. Diagnostic restriction enzyme sites were introduced 3 bp from the PAM sequence in intron 4 (NotI) and intron 6 (SalI), followed by insertion of loxP sequences (ATAACTTCGTATAATGTATGCTATACGAAGTTAT) in the same orientation.

Guide RNAs, donor template, and Cas9 nuclease were injected into blastocysts. Founder mice were screened by PCR and confirmed by Sanger sequencing. Heterozygous founders were crossed and maintained on a 129SVE/C57BL/6J mixed background homozygous for the *Rpe65* Leu450 allele. All animals were free of *rd1* and *rd8* mutations. Genotyping was performed using genomic DNA extracted from ear punch samples.

### Ethics statement

All animal experiments were conducted in accordance with the ARVO Statement for the Use of Animals in Vision and Ophthalmic Research and were approved by the Institutional Animal Care and Use Committee at West Virginia University. The mice used were maintained under a 12:12-h light/dark cycle (6:00 AM to 6:00 PM) with food and water provided ad libitum in the West Virginia University animal facility.

### Genotyping

Genotyping was performed by PCR amplification using Quick-Load Taq polymerase (New England Biolabs). PCR products were resolved by agarose gel electrophoresis or submitted for Sanger sequencing as indicated.

For *Rpe65* genotyping, the forward primer was 5′-TTACCAGAAATTTGGAGGGAAA-3′, and the reverse primer was 5′-CAGAGCATCTGGTTGAGAAACA-3′. PCR conditions were 95 °C for 5 min; 35 cycles of 95 °C for 30 s, 54 °C for 30 s, and 72 °C for 45 s; followed by 72 °C for 5 min. PCR products were sequenced (Psomagen).

For *Hsp90ab1* floxed alleles, primers flanking the intron 4 NotI site were forward 5′-GCAGTTTGGTGTCGGATTCT-3′ and reverse 5′-ACTGCCAAGCCCATACCAC-3′. Primers flanking the intron 6 SalI site were forward 5′-GTAGGCAGAAGCTGGAGGTG-3′ and reverse 5′-CAATGCCCTGAATTCCAACT-3′. PCR conditions were 95 °C for 1 min; 35 cycles of 95 °C for 30 s, 60 °C for 30 s, and 72 °C for 30 s; followed by 72 °C for 5 min.

Cre recombinase genotyping was performed by multiplex PCR using primers forward 5′-CCTGGAAAATGCTTCTGTCCG-3′ and reverse 5′-CAGGGTGTTATAAGCAATCCC-3′, with amplification control primers forward 5′-CAAATGTTGCTTGTCTGGTG-3′ and reverse 5′-GTCAGTCGAGTGCACAGTTT-3′.

*Hsp90aa1* KO genotyping was performed using multiplex PCR. Primer set 1 amplified both WT (820 bp) and KO (336 bp) alleles using forward 5′-CAATTGTAGGGGGTGTCTGG-3′ and reverse 5′-TCCTCCTCTTCTTCATCAGAGC-3′. Primer set 2 amplified WT alleles using forward 5′-TTTGTGGGGAAGGTTAGCTG-3′ and reverse 5′-TGGGATAGCCAATGAACTGA-3′. PCR conditions were 95 °C for 1 min; 35 cycles of 95 °C for 30 s, 60 °C for 30 s, and 72 °C for 3 min; followed by 72 °C for 5 min.

### Electroretinogram (ERG)

After >2 h of dark adaptation, mice were anesthetized with 2.0% isoflurane and oxygen at a flow rate of 2.5 L/min for 10 min. Pupils were topically dilated with a 1:1 mixture of tropicamide: phenylephrine hydrochloride. Mice were placed on a heated platform and maintained under 1.5% isoflurane/oxygen mixture (2.5 L/min) delivered through a nose cone. A reference electrode was placed subcutaneously in the scalp, and ERG responses were recorded from both eyes with silver wire electrodes placed on the corneal surface with hypromellose solution (2% in PBS) (Gonioscopic Prism Solution; Wilson Ophthalmic).

Rod-dominated responses (scotopic) were elicited in the dark with increasing intensities of LED white light flashes. Light-adapted cone responses (photopic) were recorded in the presence of a 30 cd/m^2^ rod-saturating background light. Recordings were obtained using the UTAS Visual Diagnostic System (LKC Technologies) with EMWIN software.

### Immunohistochemistry and histology

Eyes were enucleated and fixed in 4% paraformaldehyde (PFA) in PBS (16% PFA solution, EM grade, Electron Microscopy Sciences). After 15 min, the cornea was removed, and fixation continued for 1 h 45 min. Eyes were washed in PBS and cryoprotected in sucrose gradients (7.5%, 15%, and 22%) for at least 2 h on a nutator at 4 °C. Following sucrose incubation, eyecups were placed in a 1:1 ratio mixture of 22% sucrose and Cryo Optimal Cutting Temperature Compound (Sakura) for 1 h. The lens was removed, and the eyecup was flash-frozen in Optimal Cutting Temperature.

Sections (16 μm) were cut using CryoStar NX50 (Epredia), mounted on Superfrost Plus slides (Thermo Fisher Scientific), and stored at −20 °C. Sections were washed with PBS, blocked in buffer (PBS, 5% goat serum, 0.5% Triton X-100, and 0.05% sodium azide) for 1 h at RT, and incubated overnight at 4 °C with primary antibodies (Table S1). After washing, sections were incubated with Alexa Fluor 488 or 568-conjugated secondary antibodies (Invitrogen) for 2 h, washed, and mounted using ProLong Gold antifade reagent (Thermo Fisher Scientific). Images were acquired using a Nikon Eclipse Ti2 Inverted Confocal Microscope and processed using NIS Elements and FIJI-ImageJ software.

### Retinal histology-H&E

Eyes were fixed in Z-fixative and processed for paraffin embedding and H&E staining (Excalibur Pathology). Images were acquired using a Nikon brightfield microscope. Outer nuclear layer nuclei were quantified at 12 equidistant locations per retina, averaged across three technical and three biological replicates.

### Immunoblotting

Mice were euthanized by CO_2_ inhalation followed by cervical dislocation. Retinas were dissected, flash-frozen, and lysed in buffer (0.1% Triton X-100, 50 mM Tris-HCl, pH 7.5, 300 mM NaCl, and 5 mM EDTA) containing a protease inhibitor cocktail (Roche). Cellular debris was cleared by centrifuging lysates at 12,000 × *g* for 30 s at 4 °C. Samples were mixed with Laemmli buffer, boiled for 5 min, and separated by SDS-PAGE. Equal amounts (50 μg) were loaded per lane.

Proteins were transferred to polyvinylidene difluoride membrane (Immobilon-FL) and stained with a total protein stain (LI-COR). Membranes were blocked (Odyssey Blocking Buffer; LI-COR) for 30 min at RT, followed by overnight incubation with primary antibodies (Table S1) at 4 °C on a rotator. After washing 3 times in PBS-T (1 × PBS with 0.1% Tween 20) for 5 min each at RT, the membrane was incubated with goat anti-rabbit or anti-mouse Alexa Fluor 488, 680, or 800 secondary antibodies (Invitrogen, 1:50,000 dilution) for 30 min at RT. After three 5-min washes each with PBS-T, the membranes were imaged using a Typhoon Imaging System. Densitometry was performed using ImageJ and was normalized to total protein stain and GAPDH.

### TEM

TEM was performed as described earlier (49) Briefly, mice were euthanized, and eyes were enucleated and fixed (2% PFA, 2.5% glutaraldehyde, and 100 mM cacodylate). After fixation, the eyes were transferred to a Petri dish containing a drop of 7% sucrose buffer, and the cornea and lens were removed. The eyecups were then returned to the glass vial containing the fixative for 48 h with gentle rolling, after which they were cut into smaller pieces. The pieces were washed with an excess of 0.1 M cacodylate buffer, then incubated with 2% osmium tetroxide in 0.1 M cacodylate buffer for 1 h on ice. The samples were washed with water and incubated in 1% uranyl acetate at 4 °C. The next day, samples were dehydrated in an ethanol series, processed, and embedded in resin. The samples are then cut with a LEICA EM UC7 ultramicrotome to obtain 70 nm sections. The sections are post-stained with Reynold's lead citrate and imaged on a JEOL JEM-1400 transmission electron microscope.

### Flatmount

Eyes were enucleated and marked for orientation using a 25-gauge needle. The eyes were fixed in 4% PFA for 15 min at RT. A small incision toward the optic nerve was made at the location of the needle poke to maintain orientation. The cornea and the lens were removed; the eye cup was fixed in 4% PFA for an additional 30 min. Subsequently, the optic nerve, sclera, and RPE were separated from the retina. Isolated retinas were washed three times with 1X PBS for 5 min each, followed by a 4 h blocking step at RT in 1X PBST. The retinas were incubated overnight at 4 °C with primary antibodies at 1:500 in blocking buffer, then washed three times with 1X PBS for 30 min each. The secondary antibody (anti-mouse Alexa Fluor 488) was applied at a 1:500 dilution and incubated for 4 h at RT. The retinas were then washed three times for 30 min each with 1X PBST. After washing, the retinas were butterfly-cut and mounted photoreceptor side up onto microscope slides. The stained butterfly-cut retinas were mounted with ProLong Gold (Life Technologies), and coverslips were added and sealed with clear nail polish. Once dried, slides were imaged using a Nikon spinning-disk confocal microscope.

### Statistics and reproducibility

Age-matched male and female mice were used. Statistical analyses were performed using GraphPad Prism v10.2.3. Data are presented as mean ± SEM or mean ± SD. Statistical significance was assessed using unpaired *t*-tests or one- or two-way ANOVA with appropriate *post hoc* comparisons. Sample sizes are indicated in the figure legends.

## Data availability

All data supporting the findings of this study are contained within the article and Supporting Information.

## Supporting information

This article contains supporting information.

## Conflict of interest

The authors declare that they have no conflicts of interest with the contents of this article.
