# Supplementary material for: Cytosolic heat shock protein 90 is required for photoreceptor outer segment development and vision
Source: J Biol Chem. 2026 May 24;302(7):113192. doi: 10.1016/j.jbc.2026.113192 (PMC13315092; doi:10.1016/j.jbc.2026.113192)
Supplement: Supporting Tables and Figures [file mmc1.pdf]

Title: Cytosolic Heat Shock Protein 90 is Required for Photoreceptor Outer Segment Development and Vision.

Authors: Hunter L. Aliff [1], Alexis B. Crockett [3], Daniella Munezero [2], Chyanne Reid [1], Hayley G. Bockius [1], Sierra G. Kuzak [1], Scott Rhodes [4], Thamaraiselvi Saravanan [4], and Visvanathan Ramamurthy [1,4]

Supporting Information

Table S1: Antibody list

Fig. S1. Cre recombinase activity in *Crx*-Cre driver lines.

Fig. S2. HSP90β is dispensable for the localization of photoreceptor outer segment proteins.

Fig. S3. Cone density is preserved in the retina lacking HSP90β.

Fig. S4. Photoreceptor function is preserved in BAC-*Crx*-Cre: HSP90β$^{-/-}$ mice.

Fig. S5. Broad Cre activity in 12-kb Crx-Cre mice leads to combined loss of cytosolic HSP90 paralogs and systemic defects.

Fig. S6. Uncropped immunoblots corresponding to Fig. 5.

Fig. S7. Uncropped immunoblots corresponding to Fig. 10.

Table S1

Antibody Name	RRID:	Conc: WB	Conc: IHC
Heat Shock Protein ab1 90kD (HSP90β)	AB_2533349	1:1000	1:500
Heat Shock Protein aa1 90kD (HSP90α)	AB_2120934	1:1000	1:500
Cone Arrestin (Arr4)	AB_1163387	N/A	1:1000
Peanut Agglutinin (PNA)	AB_2336642	N/A	1:1000
Opsin Red/Green (M-Opsin)	AB_177456	N/A	1:1000
Opsin Blue (S-Opsin)	AB_177457	N/A	1:1000
ADP-ribosylation factor-like protein 13B (ARL13B)	AB_11000053	1:1000	1:1000
Retinitis Pigmentosa 1 protein (RP1)	AB_10754493	N/A	1:250
Centrin-2	AB_2077383	N/A	1:500
Phosphodiesterase 6 β (PDE6β)	AB_2619668	1:1000	1:1000
Peripherin 2 (PRPH2)	AB_10665364	1:1000	1:1000
Rhodopsin (4D2)	AB_98887	1:1000	1:1000
CD133 (E5E2H) (PROM1) Rabbit Monoclonal	#48082	1:1000	1:1000
Cone Phosphodiesterase 6 α' (PDE6α') 3184p	custom made	1:1000	1:1000
Cone Transducin (GNAT2)	AB_10858788	1:1000	1:1000

Supporting Figure S1

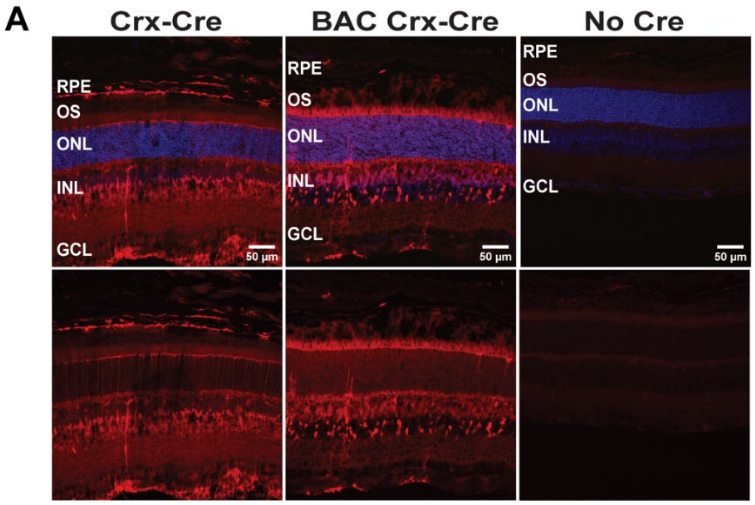

Fig. S1. Cre recombinase activity in *Crx*-Cre driver lines.

Immunohistochemistry of retinal cross-sections from mice crossed with the Ai9 tdTomato Cre reporter line at postnatal day 30 (P30). tdTomato fluorescence (red) indicates Cre activity; nuclei are labeled with DAPI (blue). Retinas from 12-kb *Crx*-Cre, 219-kb BAC-*Crx*-Cre, and Cre-negative control mice are shown.

GCL, ganglion cell layer; INL, inner nuclear layer; ONL, outer nuclear layer; OS, outer segments. Scale bar, 50 µm.

Supporting Figure S2

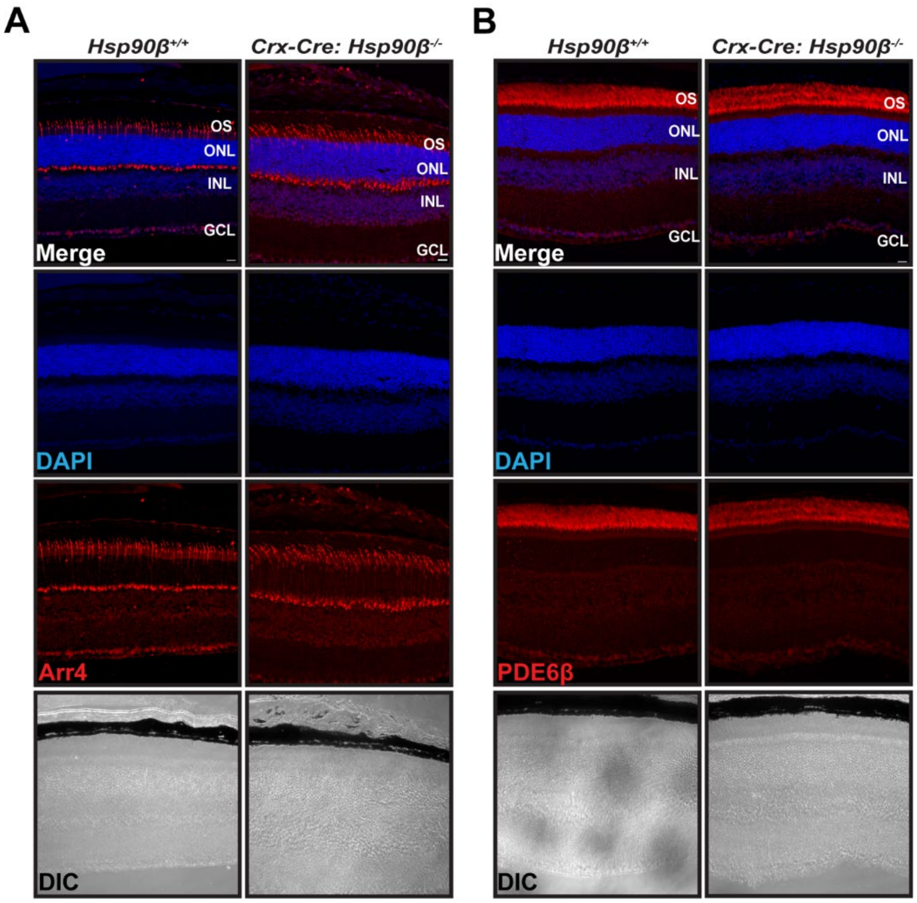

Fig. S2. HSP90β is dispensable for the localization of photoreceptor outer segment proteins.

(A) Immunohistochemistry of retinal cross sections from HSP90β$^{+/+}$ control and *Crx*-Cre: HSP90β$^{-/-}$ mice at postnatal day 300 (P300) stained for Arr4 (red) and DAPI (blue).

(B) Immunohistochemistry of retinal cross sections from the same genotypes at P300 stained for PDE6β (red) and DAPI (blue).

GCL, ganglion cell layer; INL, inner nuclear layer; ONL, outer nuclear layer; OS, outer segments

Supporting Figure S3

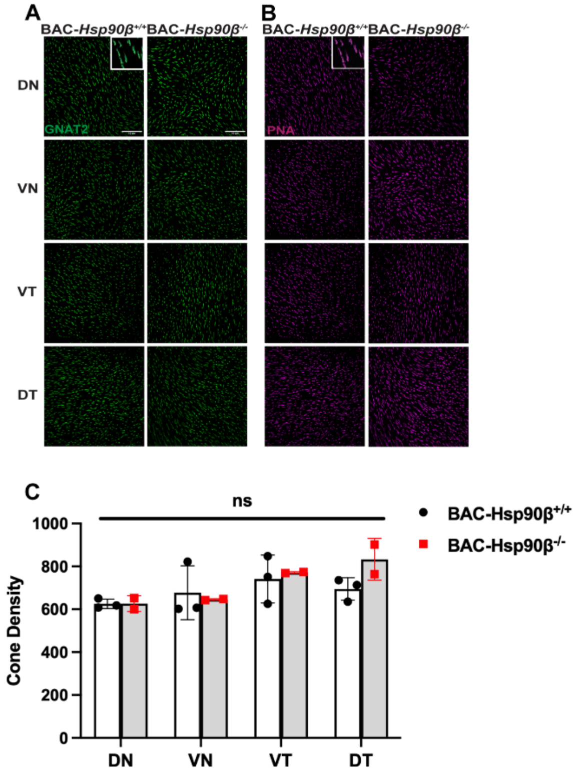

Fig. S3. Cone density is preserved in the retina lacking HSP90β.

Immunostaining of retinal flatmounts from HSP90β+/+ control and BAC-Crx-Cre: HSP90β$^{-/-}$ mice at postnatal day 60 (P60).

(A) Cone transducin α-subunit (GNAT2, green)

(B) peanut-agglutinin (PNA, magenta).

(C) Quantification of cone density in each quadrant of the flatmount (cones per 0.75mm^2). Black, HSP90β$^{+/+}$, Grey, BAC-*Crx*-Cre: HSP90β$^{-/-}$; n=3.

Data represent mean ± SEM. ns, not significant (P > 0.05). DN, dorsal nasal; VN, ventral nasal; VT, ventral temporal; DT, dorsal temporal.

Scale bar, 50 μm

Supporting Figure S4

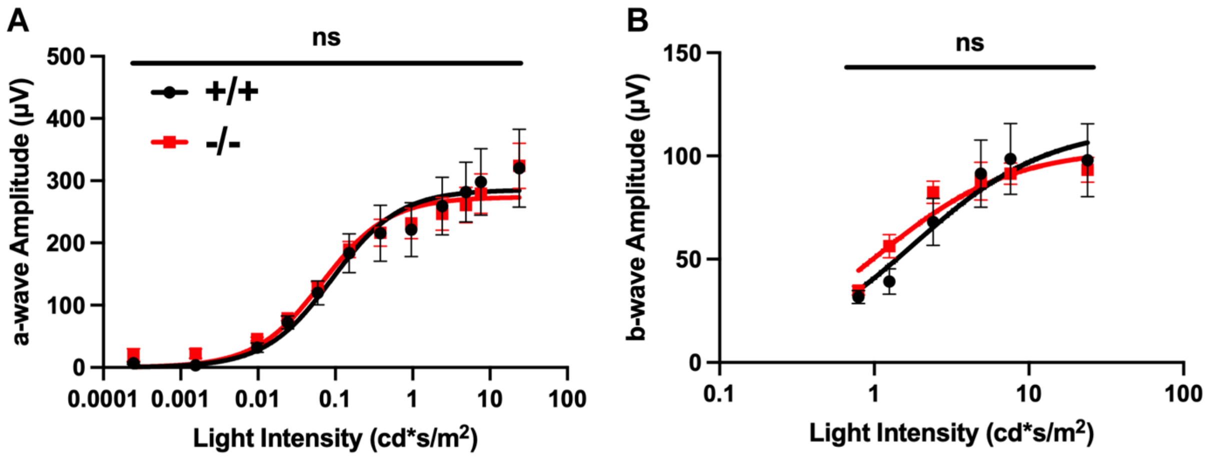

Fig. S4. Photoreceptor function is preserved in BAC-*Crx*-Cre: HSP90β$^{-/-}$ mice.

(A) Scotopic a-wave sensitivity curves from HSP90β$^{+/+}$ control (black, +/+) and BAC-*Crx*-Cre: HSP90β$^{-/-}$ (red, -/-) mice at >P60; n = 3.

(B) Photopic b-wave sensitivity curves recorded from the same genotypes at >P60; n = 3. Data represent mean ± SEM. ns, not significant (P > 0.05).

Supporting Figure 5

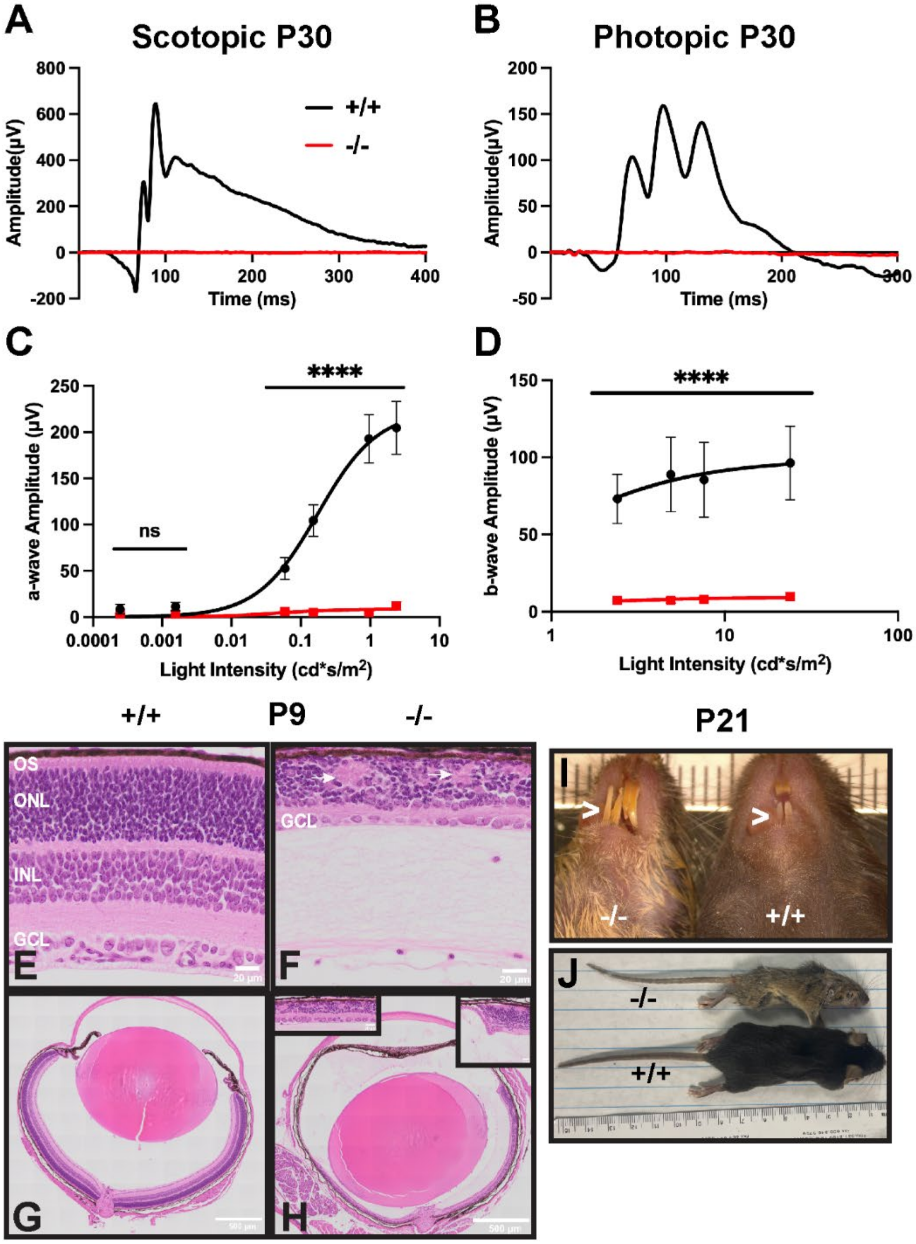

Fig. S5. Broad Cre activity in 12-kb Crx-Cre mice leads to combined loss of cytosolic HSP90 paralogs and systemic defects.

(A) Representative scotopic ERG responses recorded from HSP90$^{+/+}$ control (black, +/+) and *Crx*-Cre: HSP90$^{-/-}$ (red, -/-) postnatal day 30 (P30) at −0.1 log cd*s/m^2.

(B) Representative photopic ERG responses recorded from Hsp90$^{+/+}$ (black, +/+) and *Crx*-Cre: Hsp90$^{-/-}$ (red, -/-) P30 at 4.8 log cd*s/m^2.

(C) Scotopic a-wave sensitivity curves from HSP90$^{+/+}$ control (black, +/+) and *Crx*-Cre: HSP90$^{-/-}$ (red, -/-) mice at P30.

(D) Photopic b-wave sensitivity curves from the same genotypes at P30; n = 3.

Data represent mean ± SEM. ns, not significant ($P > 0.05$); *$P < 0.05$; ****$P < 0.0001$;

(E, F) H&E stained retinal cross sections from HSP90$^{+/+}$ (+/+) **(E)** and *Crx*-Cre: HSP90$^{-/-}$ (-/-) **(F)** at P9. Arrows in Panel F point to the rosettes observed in the ONL.

(G, H) H&E stained whole-eye cross sections from HSP90$^{+/+}$ (+/+) **(G)** and *Crx*-Cre: HSP90$^{-/-}$ (-/-) **(H)** at P9.

(I) Light microscopy of incisors from P21 HSP90$^{+/+}$ (+/+) and *Crx*-Cre: HSP90$^{-/-}$ mice(-/-).

(J) Body size comparison of HSP90$^{+/+}$ (+/+) and *Crx*-Cre: HSP90$^{-/-}$ mice (-/-).

Supporting Figure S6

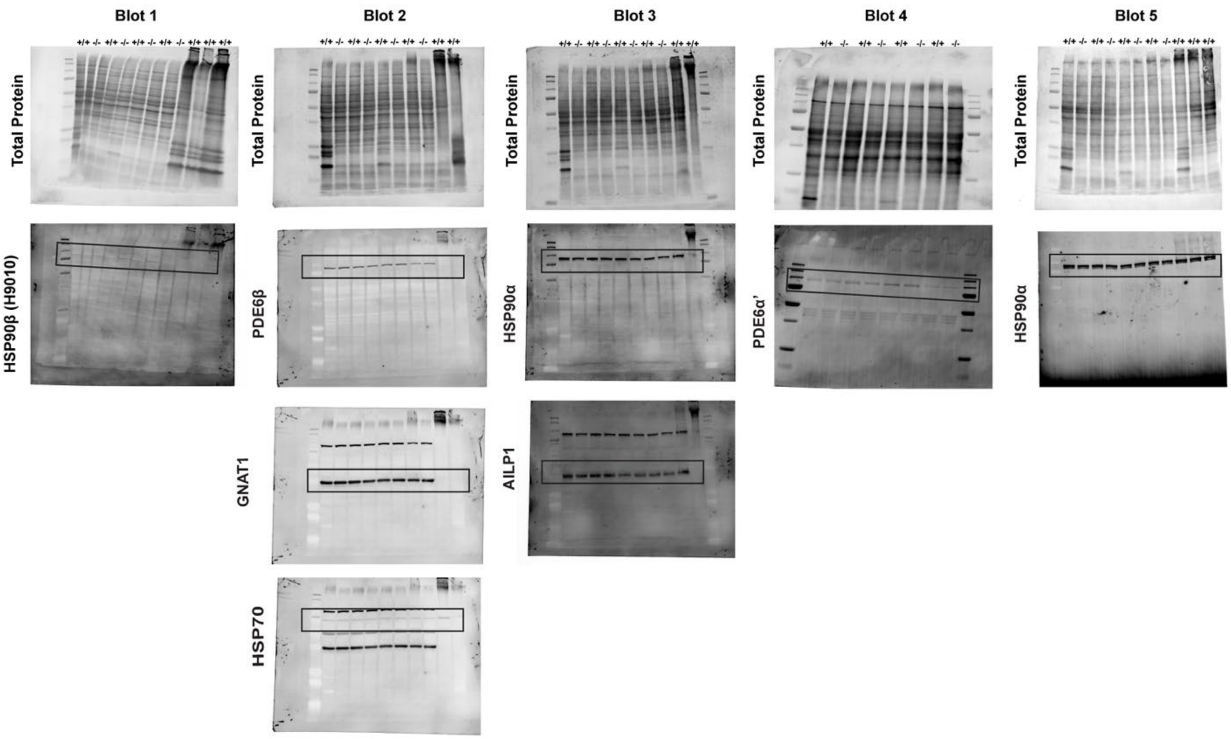

Fig. S6. Uncropped immunoblots corresponding to Fig. 5.

Immunoblot analysis of PDE6β, GNAT1, HSP70, and HSP90α in retinal lysates from HSP90β$^{+/+}$ (+/+) and *Crx*-Cre: HSP90β$^{-/-}$ (-/-) mice at postnatal day 300 (P300). Total protein staining as a loading control. (n=4).

Supporting Figure S7

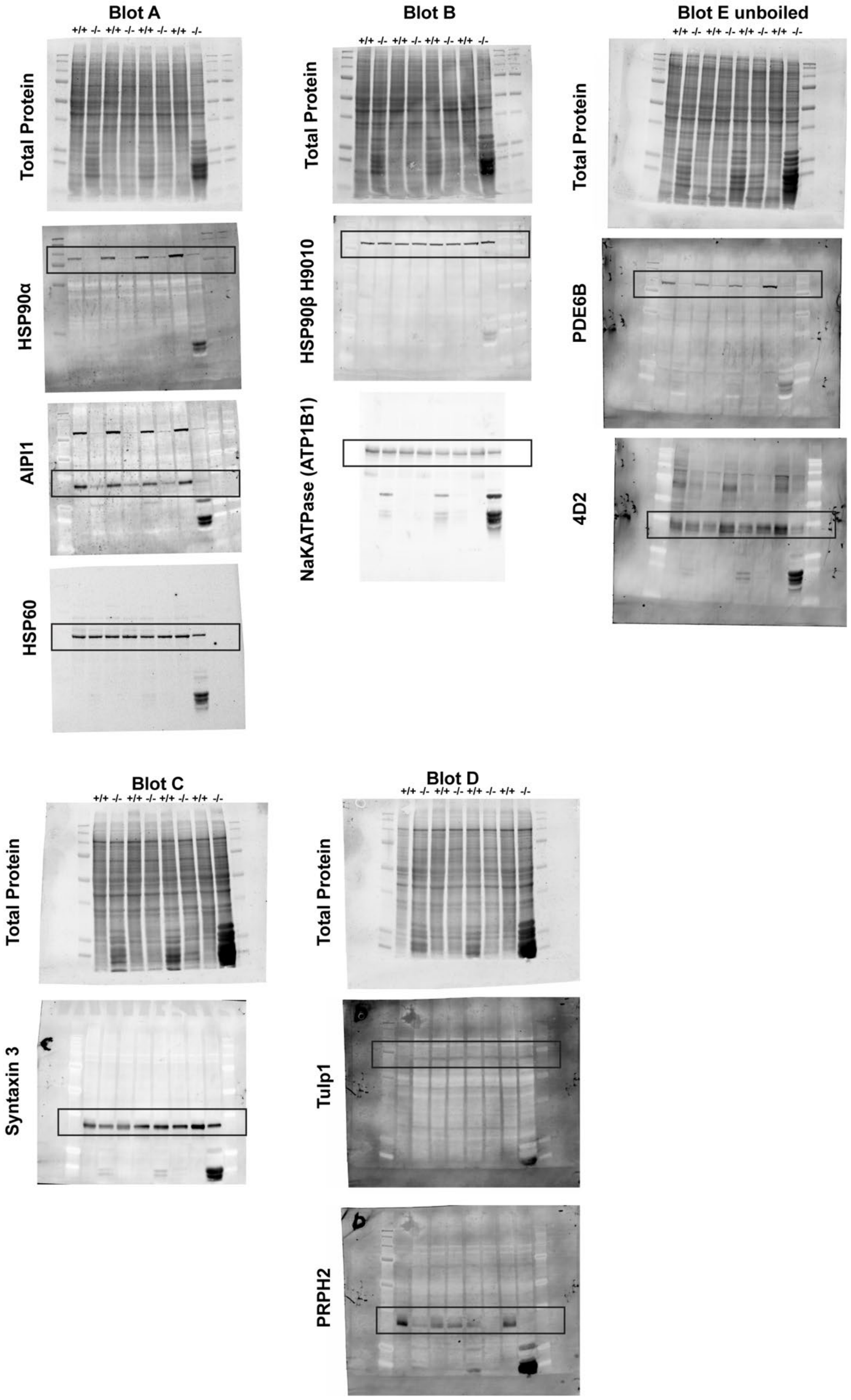

Fig. S7. Uncropped immunoblots corresponding to Fig. 12.

Immunoblot analysis of HSP90α, AIPL1, HSP90β, HSP60, ATP1B1, PDE6β, Rhodopsin, TULP1, STX3 and PRPH2 in retinal lysates from HSP90$^{+/+}$ (+/+) and *Crx*-Cre: HSP90$^{-/-}$ (-/-) mice at P9. Total protein staining was used as a loading control.
